# *Phleum pratense* pollen-derived di-galactosyldiacylglycerols promote pro-allergic responses in mice

**DOI:** 10.3389/fimmu.2025.1532773

**Published:** 2025-06-17

**Authors:** Nestor González Roldán, Lars P. Lunding, Yukari Fujimoto, Sylvia Düpow, Dominik Schwudke, Michael Wegmann, Katarzyna A. Duda

**Affiliations:** ^1^ Research Group of Biofunctional Metabolites and Structures, Research Center Borstel, Leibniz Lung Center, Research Center Borstel, Airway Research North (ARCN), German Center for Lung Research (DZL), Borstel, Germany; ^2^ Research Group of Lung Immunology, Research Center Borstel, Leibniz Lung Center, Research Center Borstel, Airway Research North (ARCN), German Center for Lung Research (DZL), Borstel, Germany; ^3^ Laboratory of Biomolecular Chemistry, Department of Chemistry, Faculty of Science and Technology, Keio University, Yokohama, Japan; ^4^ Research Group of Bioanalytical Chemistry, Research Center Borstel, Leibniz Lung Center, Research Center Borstel, Airway Research North (ARCN), German Center for Lung Research (DZL), Borstel, Germany; ^5^ Thematic Translational Unit Tuberculosis, German Center for Infection Research (DZIF), Partner Site Hamburg-Lübeck-Borstel-Riems, Hamburg, Germany

**Keywords:** airway inflammation, allergy, glycerolipids, grass pollen, structure-function

## Abstract

**Introduction:**

Grass pollen triggers nearly 30% of bronchial allergic asthma cases. While most Q8 research focuses on pollen allergens, pollen lipids may also influence allergic reactions. Previous studies demonstrated that Timothy grass (TG, Phleum pratense) lipids, such as phytoprostanes, can activate immune cells, promoting pro-allergic responses. However, the role of water-insoluble pollen glycolipids in allergic airway inflammation remains unclear. Thus, this study aimed to isolate and characterize glycolipids from TG pollen and evaluate their bioactivity in allergic airway inflammation.

**Methods:**

Lipids were extracted from the water-insoluble pollen fraction, separated by silica gel, and fractionated by HPLC. GC-MS, HR ESI-MS, and NMR confirmed the presence of di-galactosyldiacylglycerol (DGDG). The biological activity of fractions containing DGDG (DGDG-3 and DGDG-4) and synthetic DGDG variants was tested in vitro in murine and human cell systems and in vivo in mice.

**Results:**

Fraction 4 induced strong proliferation of murine NKT cells and upregulated CD69 expression in human NKT cells. Synthetic DGDG variants (DGDG-1, DGDG-2, and DGDG-3) with defined acylation profiles stimulated robust NKT-cell proliferation, with DGDG-2 and DGDG-3 increasing IL-13 production, one of the key Th2 cytokines. In vivo, only these variants caused lung inflammation marked by eosinophil infiltration but did not increase airway resistance.

**Discussion:**

This study reveals for the first time the structure-dependent role of DGDG of TG pollen grains in immune cell recognition in the context of allergic inflammation. Our data may pave the way for therapies targeting lipid components in combination with protein allergens.

## Introduction

Pollen allergy, or allergic rhinitis, affects millions globally, with an estimated prevalence of 10-30% worldwide, rising to as much as 40% in Europe and North America (1, 2). Allergic rhinitis is closely linked to asthma, as approximately 20%–30% of those with allergic rhinitis also experience asthma symptoms, significantly contributing to the global disease burden (2, 3). Grass pollen is one of the most common and potent airborne allergens, triggering allergic reactions in many sensitized individuals and exacerbating allergic rhinitis and asthma (4). Due to its widespread presence in temperate regions and its capacity to provoke both conditions, grass pollen has become a major focus of research aimed at understanding and mitigating pollen-induced allergic diseases (5, 6).

Our previous research on lipid mediators from *Phleum pratense* found that phytoprostanes enhance allergen-specific mast cell degranulation and selectively promote the expression of the cluster of differentiation 1d (CD1d) glycolipid-presenting molecule on dendritic cells, without affecting MHC molecules. This suggests a priming effect for glycolipid presentation and a potential role for natural killer T (NKT) cells in the allergic inflammatory process (7).

NKT cells, a subset of T lymphocytes originally described by their co-expression of T- and NK-cell markers, play a critical role in modulating immune responses, including allergic inflammation. Activated by glycolipid antigens presented on CD1d by antigen-presenting cells (APCs), NKT cells rapidly release cytokines essential for immune regulation, such as IL-4 and IFN-γ (8). NKT cells contribute to allergic inflammation, initiating and amplifying allergic responses by promoting Th2-type immunity, which is central to conditions like asthma and allergic rhinitis (9). Studies have shown that upon activation, NKT cells enhance the recruitment of eosinophils and other inflammatory cells to the allergen exposure site, exacerbating airway inflammation (10). NKT cells also interact with dendritic cells to amplify allergic inflammation through glycolipid presentation, further supporting their role in driving allergic responses (11). These findings underscore the significance of NKT cells in allergic inflammation and suggest that targeting their activation could offer a therapeutic strategy for allergic diseases.

For an allergic reaction to occur, the allergen must be present, with its availability primarily determined by the concentration of airborne pollen. However, grass pollen allergens are not delivered as isolated proteins to mucosal surfaces but as part of complex particles containing pollen-associated microorganisms capable of triggering an inflammatory response and pollen-derived compounds, including carbohydrates and bioactive lipids, such as glycolipids. Previous studies indicated that pollen glycolipids can be recognized as antigens by human T cells through a CD1-dependent pathway. Phospholipids like phosphatidylcholine and phosphatidylethanolamine, extracted from cypress pollen, have been shown to stimulate T-cell proliferation in cypress-sensitive individuals, particularly a subpopulation of TCRγδ^+^ cells, and to a lesser extent, NKT cells. In addition, these T-cell populations promoted IgE production by secreting IL-4, IFN-γ, IL-10, and TGF-β. These responses were most prominent in allergic individuals during the pollen season (12). Furthermore, dendritic cells exposed to olive pollen lipids upregulated CD1d and CD86 while downregulating CD1a, facilitating lipid recognition and activation of invariant invariant natural killer T (iNKT) cells (13).

This study addresses an important question: Despite widespread exposure to environmental allergens, only a minority of individuals—typically between 20% and 40%, depending on population and allergen—develop allergic sensitization (1). This observation suggests that allergen exposure alone is not sufficient to trigger an allergic response. In addition to the known effect of epithelial tissue disruption by proteases released from pollen grains upon hydration (14), our previous results support the hypothesis that sensitization may require both contact with allergen and concurrent exposure to lipid adjuvants with specific chemical structures (7). These lipids likely function as immune modulators, enhancing the recognition and response to allergens, ultimately contributing to the development of allergic sensitization. Elucidating the mechanism beyond the lipid–allergen interaction could open new therapeutic strategies aimed at targeting lipid-adjuvant interactions to prevent or reduce allergic diseases. While prior research has recognized the role of lipid mediators in immune modulation, the contribution of glycolipids of pollen grains to allergic responses via recognition by NKT cells, particularly within the context of airway inflammation, has not yet been fully explored. In this study, we aimed to fill this gap by targeting the isolation and characterization of glycolipids from TG pollen and assessing their potential role in promoting allergic inflammation.

## Materials and methods

### Isolation and purification of DGDG

Pollen grains from Timothy grass (TG, *Phleum pratense*) were purchased from Greer^®^ and kept at – 20°C prior to use. After removal of water-soluble compounds (water extraction in an ultrasonic bath followed by centrifugation [30 min, 3,900×*g*, 20°C]), grains were further processed with chloroform/methanol/water extraction utilizing a Branson Sonifier 250 for 20 min on ice (15). The chloroform phase containing lipids was sterile filtered (0.2 µm), dried, and further fractionated on the silica gel 60 column (10 cm × 1 cm; 0.04–0.063 mm, Merck Millipore, Molsheim, France) with increasing volumes of methanol. Based on the GC-MS chemical composition and *in vitro* experiment results, the fraction 4 CHCl_3_/MeOH, 90/10, v/v, was further separated on a HPLC system (Gilson, Middleton, Winsconsin, USA) equipped with a Kromasil 100 C18, 5 µm, 250 × 10 mm (MZ-Analysentechnik GmbH, Mainz, Germany) column with an eluent A CHCl_3_/MeOH/H_2_O, 240/1,140/620 v/v/v, containing 10 mM NH4-acetate and eluent B CHCl_3_/MeOH 1,400/600 v/v, containing 50 mM NH4-acetate. The following gradient steps were applied: 1%–10% B, 120 min; 10%–100% B, 30 min; isocratic 100% B, 30 min. The flow rate was 2 mL/min, and the eluting material was detected with a light scattering Sedex 85 detector (Sedere LT-ELSD).

**Figure 1:**
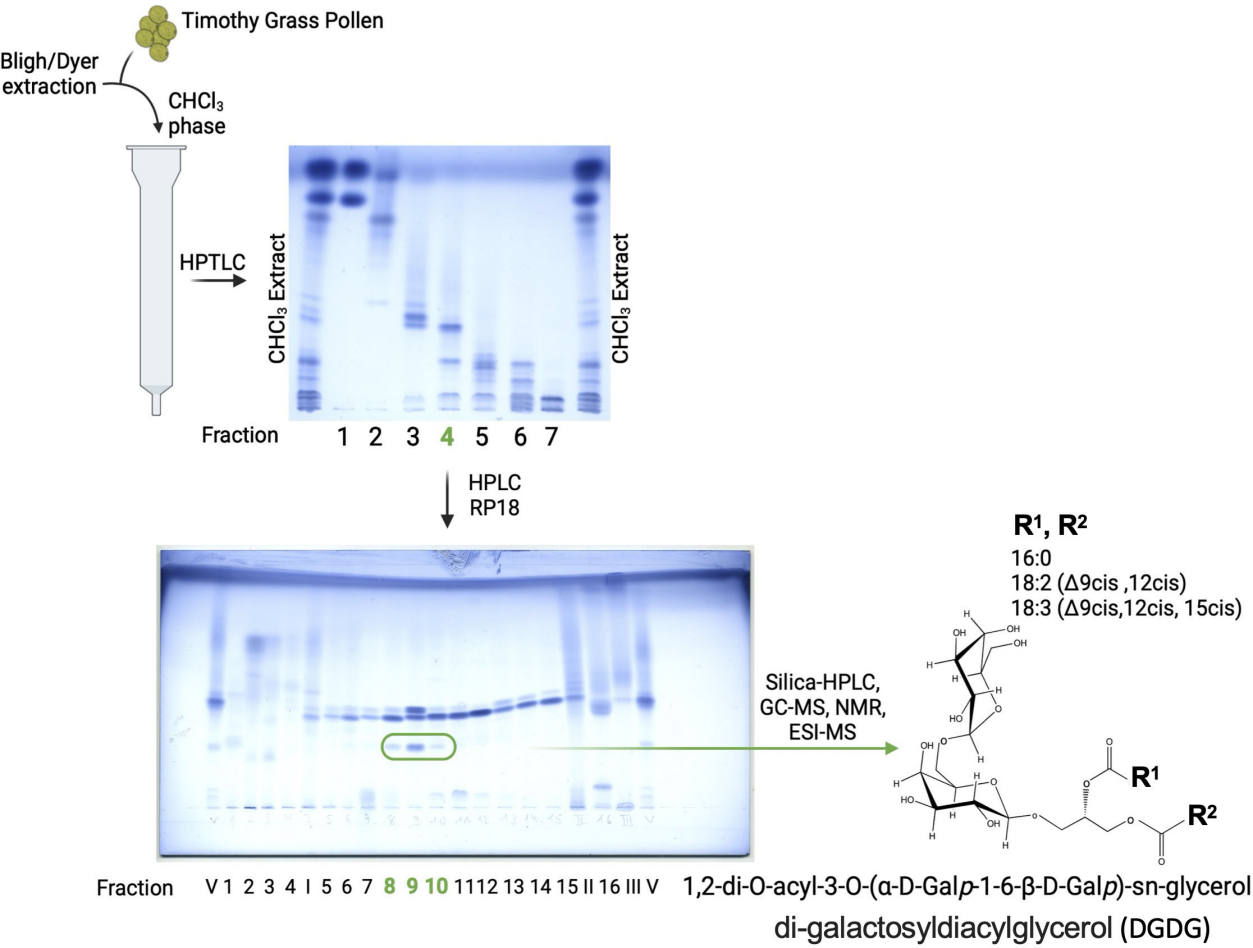
Isolation and chemical characterization of DGDG from grass pollen. Further purification steps, as described in the Materials and methods section, of fraction 4 resulted in pure DGDG molecules with a differential fatty acid profile, as indicated by GC-MS, ESI-MS, and NMR analyses.

Obtained fractions were analyzed by GC-MS, and fractions 8–10 after RP18-HPLC were subsequently separated utilizing a silica-HPLC system with a nucleosil column (100–5, 5 µm, 250 × 4.6 mm, M&N) in *n*-hexane as buffer A and CHCl_3_/MeOH 7/3 v/v as buffer B in an isocratic run of 50% B in 50 min as eluent. The flow rate was 0.5 mL/min, and the eluting material was detected with a light scattering Sedex 55 detector (Sedere LT-ELSD), leading to the isolation of pure DGDG molecules as depicted in **Figure 1**.

At each purification step, the samples have been visualized on the thin-layer chromatography (HPTLC, Merck 10 cm × 10 cm) with running buffer CHCl_3_/MeOH/H_2_O, 30/7/0.5 v/v/v, and stained for lipids with ammonium molybdate-perchloric acid-HCl reagent (16).

### Compositional analyses of DGDG

To determine the chemical composition of the fractions obtained and to identify those containing glycolipids after each step of purification, the samples were analyzed by GC-MS either after hydrolysis with 2 M HCl/MeOH (1 h, 85°C) followed by a peracetylation (10 min, 85°C) or with 2 M NaOH followed by a peracetylation (10 min, 85°C) and when needed compared to external standards of O-Me-GlcAc and O-MeGalAc.

The absolute configuration was determined by GLC of the acetylated *O*-[(R)-(−)-2-butanol] glycoside after methanolysis (2 M HCl/MeOH, 85°C, 45 min), butanolysis (2 M HCl/(R)-(−)-2-butanol, 65°C, 4 h), and peracetylation (85°C, 10 min), by comparison with authentic standards (17).

For the exact determination of the position of the double bond in 18:2 and 18:3 in DGDG, fatty acids of DGDG were derivatized to 3-pyridylcarbinol (“picolinyl”) esters (18). The fatty acids were released (1 M NaOH–MeOH, 1 h, 85°C), recovered in CHCl3, treated with trifluoroacetic anhydride (1 h, 50°C) and subsequently with 20% (w/v) 3-pyridinemethanol solution in tetrahydrofuran (1 h, 50°C) and injected in GC/MS.

GC-MS measurements were performed on an Agilent Technologies (Santa Clara, California, USA) 7890A gas chromatograph equipped with a dimethylpolysiloxane column (Agilent, HP Ultra 1; 12 m × 0.2 mm × 0.33 µm film thickness) and a 5975C series MSD detector with electron impact ionization (EI) mode under autotune conditions at 70 eV. The temperature program was 70°C for 1.5 min, then 60°C min^−1^ to 110°C and 5°C min^−1^ to 320°C for 10 min.

### The structure of DGDG was elucidated utilizing ESI-MS and NMR

High-resolution Fourier transform ion cyclotron resonance mass spectrometry (FT-ICR MS) was performed in positive ion mode using an APEX Qe-Instrument (Bruker Daltonics, Bremen, Germany) equipped with a 7-Tesla actively shielded magnet coupled with a Triversa Nanomate (Advion Interchim, Ithaca, NY, USA) as autosampler and nano-ESI source. Samples were dissolved at a concentration of ~ 5 ng/μL in a mixture of CHCl_3_/MeOH 0.1% ammonium acetate/isopropanol 1:2:4 (v/v/v). A spray voltage of 1.1 kV and a back pressure of 1.1 psi were applied, resulting in a flow rate of approximately 300 nL/min. NMR measurements of pure, isolated DGDG were recorded in CDCl_3_/MeOH/D_2_O 40:53:7 at 27°C. Spectra were calibrated to TMS (dH 0.0, dC 0.0) at 27°C. All 1D and 2D NMR experiments—including ^1^H, ^1^H correlation spectroscopy (COSY), double-quantum-filtered correlation spectroscopy (dqf COSY), total correlation spectroscopy (TOCSY), and rotating-frame Overhauser effect spectroscopy (ROESY), as well as ^1^H, ^13^C HSQC, ^1^H, ^13^C HSQC-TOCSY, and ^1^H, ^13^C HMBC—were recorded on a Bruker DRX Avance III 700 MHz spectrometer (operating frequencies of 700.75 MHz for ^1^H NMR and 176.2 MHz for ^13^C NMR) and standard Bruker software (Bruker, Rheinstetten, Germany). COSY, TOCSY, and ROESY experiments were recorded using data sets (t1 by t2) of 4,096 by 512 points. The TOCSY experiments were carried out in the phase-sensitive mode with mixing times of 120 ms and ROESY of 300 ms. The ^1^H, ^13^C correlations measured in the ^1^H-detected mode via HMBC spectra were acquired using datasets of 4,096 by 512 points, 16 scans for each t1 value of 145 Hz and long-range proton–carbon coupling constant of 10 Hz.

### NKT cell activation

Splenocyte and/or liver cell suspensions from wild-type (WT) and CD1d^−/−^ mice, free of erythrocytes (at a concentration of 1 × 10^7^; cells/mL), were labeled with 0.5 μM CFSE (Cell Division Tracker Kit, BioLegend, San Diego, California, USA) for 8 min at room temperature. After labeling, the cells were washed, seeded at 100 µL per well, and cultured for 96 h at 37 °C with 5% CO_2_ in the presence of either αGalCer (200 ng/mL), fraction 4, and DGDG-1–5 (at concentrations of 20, 2, and 0.2 µg/mL, respectively). Stock solutions of αGalCer and fraction 4 were initially prepared in PBS containing 0.5% Tween-20. For cell stimulation, the required volume of stock was diluted into prewarmed culture medium containing 10% FCS, such that the final volume per well was 200 µL and the final concentration of Tween-20 in the culture did not exceed 0.05%. Cell proliferation was assessed by CFSE dilution through flow cytometry. Dead cells were excluded from the analysis using Zombie Aqua™ Fixable Dye, following the manufacturer’s protocol, and nonspecific antibody binding was blocked using anti-CD16/32 (clone 93, BioLegend). The cells were then stained with anti-CD45R (RA3-6B2) and anti-TCRβ (H57-597) antibodies from BioLegend, and CD1d tetramers loaded with PBS57 (provided by the NIH Tetramer Core Facility). Staining was performed at 4°C in the dark for 20 min, followed by washing with FACS buffer, fixation with BioLegend’s fixation buffer, and storage at 4°C until acquisition. Samples were acquired on a MACSQuant 16 and analyzed using the FlowLogic software (Inivai Technologies, Mentone, Victoria, Australia). IFN-γ and IL-13 in the supernatant were quantified using ELISA kits (DY485, and DY413, respectively; R&D, Minneapolis, Minnesota, USA).

### Whole human blood cryopreservation and stimulation

We utilized blood samples of healthy donors who did not suffer from allergies from the blood donation service, Research Center Borstel, Germany. The study was approved by the ethical board of the University of Lübeck (EK HL AZ 2024-583). All patients provided informed voluntary consent to participate in the study according to the Helsinki Declaration of the World Medical Association (WMA Declaration of Helsinki—Ethical Principles for Medical Research Involving Human Subjects, 2013) and personal data processing. All samples and associated data were anonymized.

Freshly collected heparinized whole blood was cooled in an ice bath for 15 min, and DMSO was slowly added under constant stirring until a final concentration of 10% was achieved. One-milliliter aliquots were transferred to cryotubes and placed into a CoolCell™ rack (Corning, Corning, New York, USA), stored at − 80°C for 24 h, and finally transferred into the vapor phase of liquid nitrogen until further use.

For stimulation of cryopreserved whole blood samples, the blood was thawed quickly in a water bath at 37°C, and the blood was transferred to 15 mL conical bottom tubes and washed twice with warm nonsupplemented Iscove's Modified Dulbecco's Medium (IMDM, Capricorn Scientific, Ebsdorfergrund, Germany). The remaining pellet was resuspended in 1 mL of X-VIVO Media (Lonza, Basel, Switzerland) containing 30 units of RNase-free DNase I (Thermo Scientific, Waltham, MA, USA and incubated at 37°C, 5% CO_2_ for 2 h. Cells were then washed twice with warm nonsupplemented IMDM and resuspended in 1 mL of X-VIVO Media, and 100 µL of the cell suspension was transferred to each well into a 96-well round-bottom plate. Cells were then left either untreated or incubated in the presence of 200 ng/mL of αGalCer or 0.5, 1, 5, or 10 µg/mL of fraction 4 for 24 h at 37°C, 5% CO_2_. After 24 h, cells were stained for 30 min at room temperature with Zombie UV™, Human TruStain FcX™, antibodies anti-human Vα24-Jα18TCR (6B11), CD8 (SK1) from BioLegend, Vδ1 TCR (TS8.2, GeneTex, Irvine, California, USA), and Vβ11TCR (C21, Beckman Coulter, Brea, California, USA), acquired in a BD LSR II and analyzed using FlowJo (Version 10, BD).

### Synthesis of DGDG analogs

**Figure 2:**
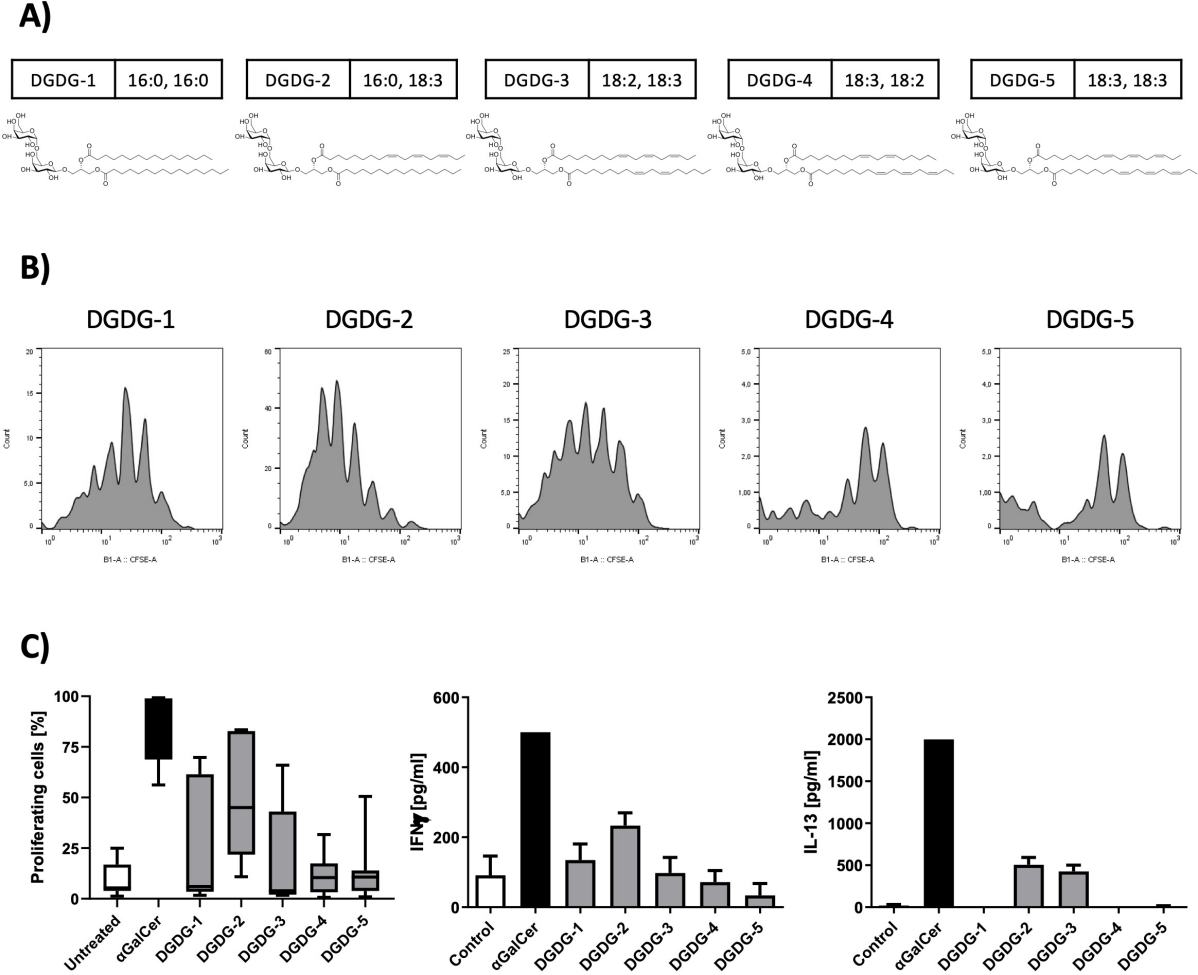
Chemical structures of the synthetic DGDG analogs and their effects on NKT cell activation and cytokine production. **(A)** The chemical structures of the synthetic DGDG compounds are shown, with the composition of the fatty acids displayed next to each compound name. **(B)** A spleen NKT cell-proliferation assay was used to assess the activity of the synthetic DGDG analogs. Representative histograms show that DGDG-1, DGDG-2, and DGDG-3 induced strong NKT cell activation, while DGDG-4 and DGDG-5 did not. αGalCer was used as a positive control, and untreated cells served as a negative control. After 96 h of culture with the compounds, cells were harvested and analyzed by flow cytometry. **(C)** The left graph depicts the percentages of proliferating NKT cells from panel B, while the middle and right graphs show the levels of IFN-γ and IL-13 in the supernatants from the same proliferation experiments, measured by ELISA. DGDG-2 and DGDG-3 selectively induced IL-13 production.

DGDG analogs (DGDG-1~5, **Figure 2A**) were synthesized based on the previously reported methods. (19) Briefly, for the divergent synthesis of the DGDG analogs with various lipid structures, a digalactosyl glycerol-containing common key intermediate was first prepared. The fatty acyl groups (combination of either C16:0, C16:0/C16:0, C18:3/C18:2, C18:3/C18:3, C18:2/C18:3, or C18:3) were then introduced to the key intermediate stepwisely, at the final stage of the synthesis, to give the target compounds, DGDG-1~5.

### *In vivo* studies and protocols

#### Animal treatment protocols

Female wild-type C57BL/6 and CD1d knockout mice (Cd1d^−/−^), aged 12 weeks, were housed under specific pathogen-free conditions, receiving food and water ad libitum. All animals were born and raised in the animal facility of the Research Center Borstel, Germany. All experimental procedures were approved by the animal ethics committee at the MLLEV, Kiel, Germany, V224-32189/2020 (56-6/20). All experiments were performed in accordance with the Geneva Convention “International Guiding Principles for Biomedical Research Involving Animals” (Geneva, 1990) and the World Medical Association Declaration of Helsinki on the Humane Treatment of Animals (2000 edition). Mice were treated intratracheally (i.t.) with glycolipid (0.1 µg/mL aGalCer; 0.4 µg/mL F4; 0.4 µg/mL DGDG1-3) dissolved in 50 µL sterile phosphate-buffered saline (PBS). All animals were killed after assessment of airway hyperresponsiveness by cervical dislocation under deep anesthesia. Sampling (serum, bronchoalveolar lavage (BAL), lung tissue) was performed 24 h after i.t. treatment. Animals for the negative control group were sham-treated with PBS (i.t.). Twelve animals per group were used.

#### Bronchoalveolar lavage and differential cell count

Lungs were lavaged with 1 mL of ice-cold PBS containing a protease inhibitor (complete; Roche, Basel, Switzerland) via a tracheal cannula. Cells were counted using a Neubauer counting chamber. Aliquots of 50 µL of the lavage fluid were cytospinned (Cytospin™; Thermo Fisher Scientific, Waltham, MA, USA), stained with Diff-Quick (Medion Diagnostic, Düdingen, Switzerland), and the cells were microscopically differentiated based on morphological criteria as previously described (20).

#### Assessment of airway hyperresponsiveness

Airway responsiveness to methacholine (acetyl-β-methylcholine chloride; Sigma-Aldrich, St. Louis, MO, USA) was invasively measured in anesthetized and ventilated mice 24 h after intratracheal treatment. This was done using FinePointe RC Units (Data Science International, St. Paul, MN, USA) to continuously monitor airway resistance (RI). The mice were anesthetized with ketamine (90 mg/kg body weight; CP-Pharma, Burgdorf, Germany) and xylazine (10 mg/kg body weight; CP-Pharma, Burgdorf, Germany), and a tracheal cannula was inserted, followed by mechanical ventilation as previously described (21). Measurements were recorded at baseline (PBS) and after exposure to increasing concentrations of aerosolized methacholine (3.125, 6.25, 12.5, 25, 50, and 100 mg/mL). After the lung function assessment, the animals were euthanized by cervical dislocation under deep anesthesia.

#### Lung histology

Mouse lungs were inflated and fixed *ex situ* by instilling 4% (w/v) phosphate-buffered paraformaldehyde under constant pressure for 20 min. The lungs were then stored at 4°C in paraformaldehyde overnight and subsequently embedded in paraffin. Lung orientation was randomized using the orientator technique (22). For lung inflammation analysis, 2 μm sections were stained with periodic acid-Schiff (PAS). Photomicrographs were captured with a digital camera (DP-25; Olympus, Tokyo, Japan) attached to a microscope (BX-51, Olympus, Tokyo, Japan) at × 20 magnification, using Olympus cell^A software.

#### Quantitative morphology

PAS was used to stain mucus in the airways. For mucus assessment, systematic uniform random samples of lung tissue were taken following standard methods (23). The surface area of mucin-containing goblet cells (Sgc) per total surface area of airway epithelial basal membrane (Sep) was determined using a computer-assisted stereology toolbox (newCAST, Visiopharm, Hoersholm, Denmark) (24, 25) according to the following formula: Sgc/Sep = ∑Igc/∑Iep, where ∑Igc is the sum of intersections of test lines with goblet cells and ∑Iep is the sum of all intersections of test lines with the epithelial basal membrane (26).

#### Assessment of cytokines

Levels of murine IFN-γ, IL-4, IL-5, IL-6, IL-10, IL-13, IL-17A, IL-33, and TNF-α in BAL were measured using MSD U-Plex Assays (Meso Scale Diagnostics, Rockville, MD, USA) according to the manufacturer’s guidelines, with detection performed using the MESO QuickPlex SQ 120MM (Meso Scale Diagnostics).

#### Reverse transcription and quantitative real-time PCR for murine samples

Ribonucleic acid (RNA) was extracted using the RNeasy^®^ Micro Kit (Qiagen, Hilden, Germany) and reverse transcribed into complementary DNA (cDNA) with the Maxima First Strand cDNA Synthesis Kit (Thermo Fisher Scientific, Waltham, MA, USA) following the manufacturer’s instructions. Real-time polymerase chain reaction (PCR) was conducted on a LightCycler 480 II instrument (Roche Applied Science, Mannheim, Germany) using the LightCycler 480 SYBR Green II Master (Roche Applied Science, Mannheim, Germany) in a total volume of 10 µL. The cycling conditions were as follows: one cycle at 95°C for 10 min, followed by 45 cycles of touchdown PCR from 63°C to 58°C for 8 s (decreasing by 0.5°C/s), and 72°C for 10 s. A standard curve was established for each primer pair using serial dilutions of cDNA. RPL-32 served as the housekeeping gene. The following primers were used: Cxcl9 (QT00097062, Qiagen, Germany)′; Ccl17 forward 5′-AATGTAGGCCGAGAGTGCTG-3′, reverse 5′-TGGCCTTCTTCACATGTTTG-3′; Ccl22 forward 5′-CAAGCCTGGCGTTGTTTTGAT-3′, reverse 5′-TAGAGGGACCAGAGCCTCAC-3′. Data were analyzed using the “advanced relative quantification” and “standard curve method”. A calibrator cDNA was included in each run to account for inter-run variability. Amplification specificity was confirmed with a melting curve analysis, and PCR products were visualized by agarose gel electrophoresis.

### Statistical analysis

The significance of differences was evaluated using one-way ANOVA. Tukey’s *post-hoc* test was employed in pairwise comparisons. GraphPad Prism 10.4.1 was used for data analysis and graph preparation. Data are presented as mean ± SEM (^∗∗∗∗^ *p* < 0.0001; ^∗∗∗^ *p* < 0.001, ^∗∗^ *p* < 0.01, and ^∗^ *p* < 0.05), for comparisons as indicated.

## Results

### Chemical composition of Timothy grass pollen

**Figure 3:**
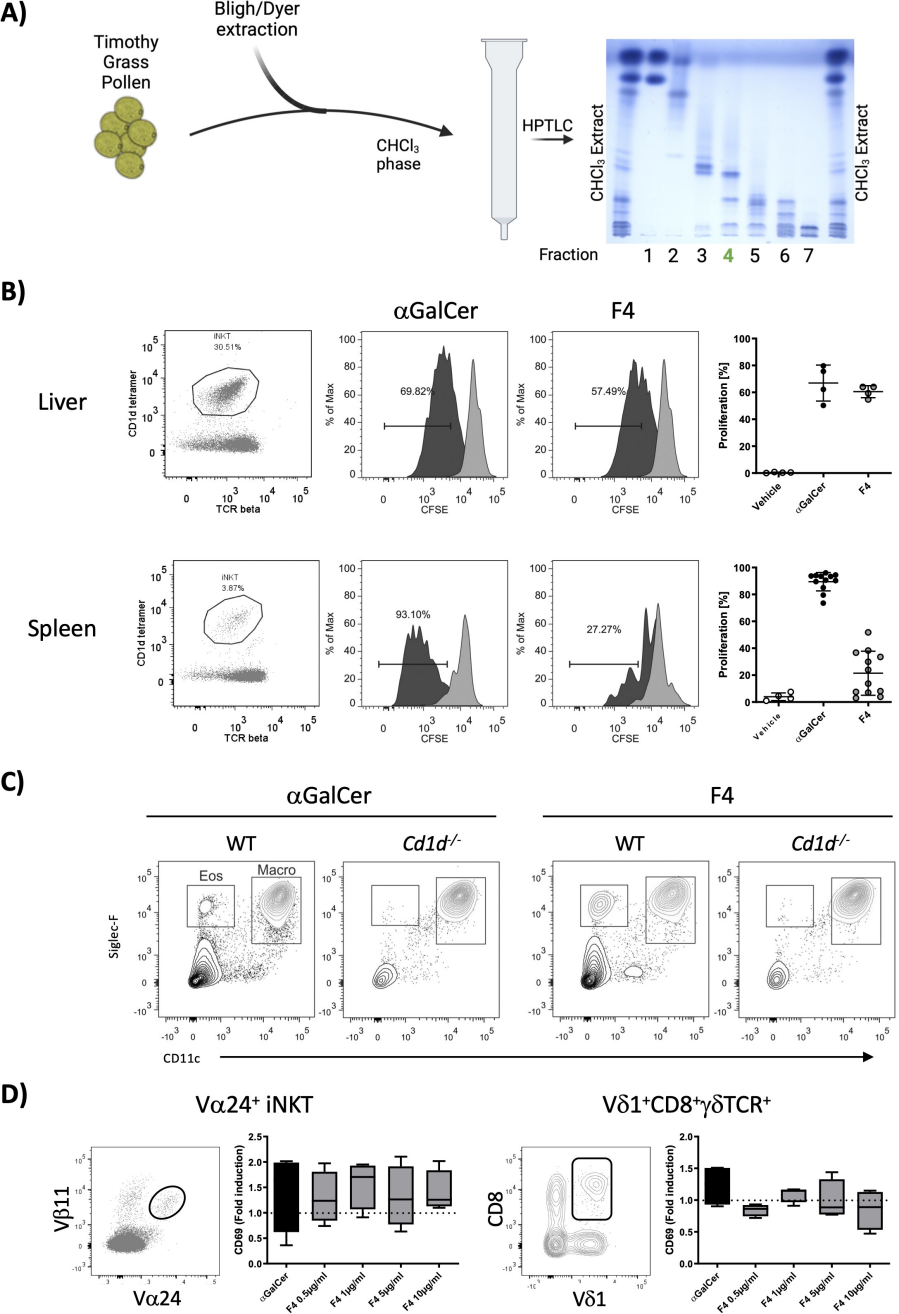
Isolation and *in vitro* characterization of fraction 4. **(A)** Timothy grass pollen was extracted with the Bligh/Dyer method. The CHCl_3_ layer was fractionated on silica gel with a polarity gradient, yielding seven fractions, which were visualized by HPTLC; fraction 4, used in further experiments, is marked in green. **(B)** Fraction 4 was tested for its ability to stimulate liver and spleen NKT cells in a cell-proliferation assay. Representative dot plots show the gated NKT-cell population, and the accompanying histograms illustrate CFSE dilution. The graphs on the right show the percentages of proliferating cells, with αGalCer serving as the positive control. Data are shown for liver NKT cells (*n* = 4) and spleen NKT cells (*n* = 12). **(C)** Wild-type and Cd1d^−/−^ mice received an intranasal dose of either 2 µg of αGalCer or 20 µg of fraction 4; bronchoalveolar-lavage cells collected 24 h later were analyzed by flow cytometry. Fraction 4 provoked marked eosinophilic infiltration, with CD11c-Siglec-F^+^ cells classified as eosinophils. **(D)** Whole blood from healthy donors was stimulated *in vitro* with either αGalCer or fraction 4. Glycolipid-reactive cells (NKT Vα24^+^Vβ11^+^ and a subpopulation of γδ T cells, Vδ1^+^CD8^+^γδTCR^+^) were analyzed for CD69 expression. Median fluorescence-intensity values were normalized to unstimulated controls. *n* = 4.

After silica gel separation, two fractions—namely fraction 3 (93/7, CHCl_3_/MeOH, v/v) and fraction 4 (90/10, CHCl_3_/MeOH, v/v)—contained hexose, glycerol (Gro), saturated fatty acids (predominantly 18:0, 16:0), unsaturated fatty acids (18:1, 18:2, 18:3), phytosphingosine, and hydroxy fatty acids, indicating the presence of glycolipids, which were our target molecules for testing activation in NKT cells (**Figure 3A**).

### Fraction 4 induces NKT cell activation and eosinophilia via CD1d-dependent mechanisms in mice and stimulates human immune cells

We assessed the biological activity of fraction 3 (not shown) and fraction 4 (**Figure 3B**) by examining NKT cell proliferation *in vitro* using erythrocyte-free cell suspensions from mouse liver and spleen. Fraction 4 induced much more robust NKT cell activation in both tissues as compared to fraction 3. To further investigate the potential of fraction 4 to elicit a response in the lungs, we administered the fraction intranasally to wild-type and Cd1d^−/−^ mice, alongside αGalCer as a control. After 24 h, BAL fluid was collected for cell content analysis. Fraction 4 induced significant eosinophilic infiltration in wild-type mice, while this response was absent in Cd1d^−/−^ mice, indicating a CD1d-dependent mechanism (**Figure 3C**). The macrophage population was minimally affected by either treatment. Additionally, to determine if fraction 4 was capable of stimulating human immune cells, we incubated whole blood from healthy donors with 20 µg of fraction 4 or αGalCer and analyzed the expression of the activation marker CD69 on NKT cells and γδT-cell subpopulations by flow cytometry. We observed a notable upregulation of CD69 on both NKT cells and a Vδ1^+^CD8^+^γδTCR^+^ cell subpopulation (**Figure 3D**), suggesting that human cells also responded to the lipid compounds in fraction 4. These findings led us to prioritize fraction 4 for further fractionation and characterization of the active galactolipids responsible for their biological activity.

### Fraction 4 induces inflammation and mucus production of the airways, similar to αGalCer

**Figure 4:**
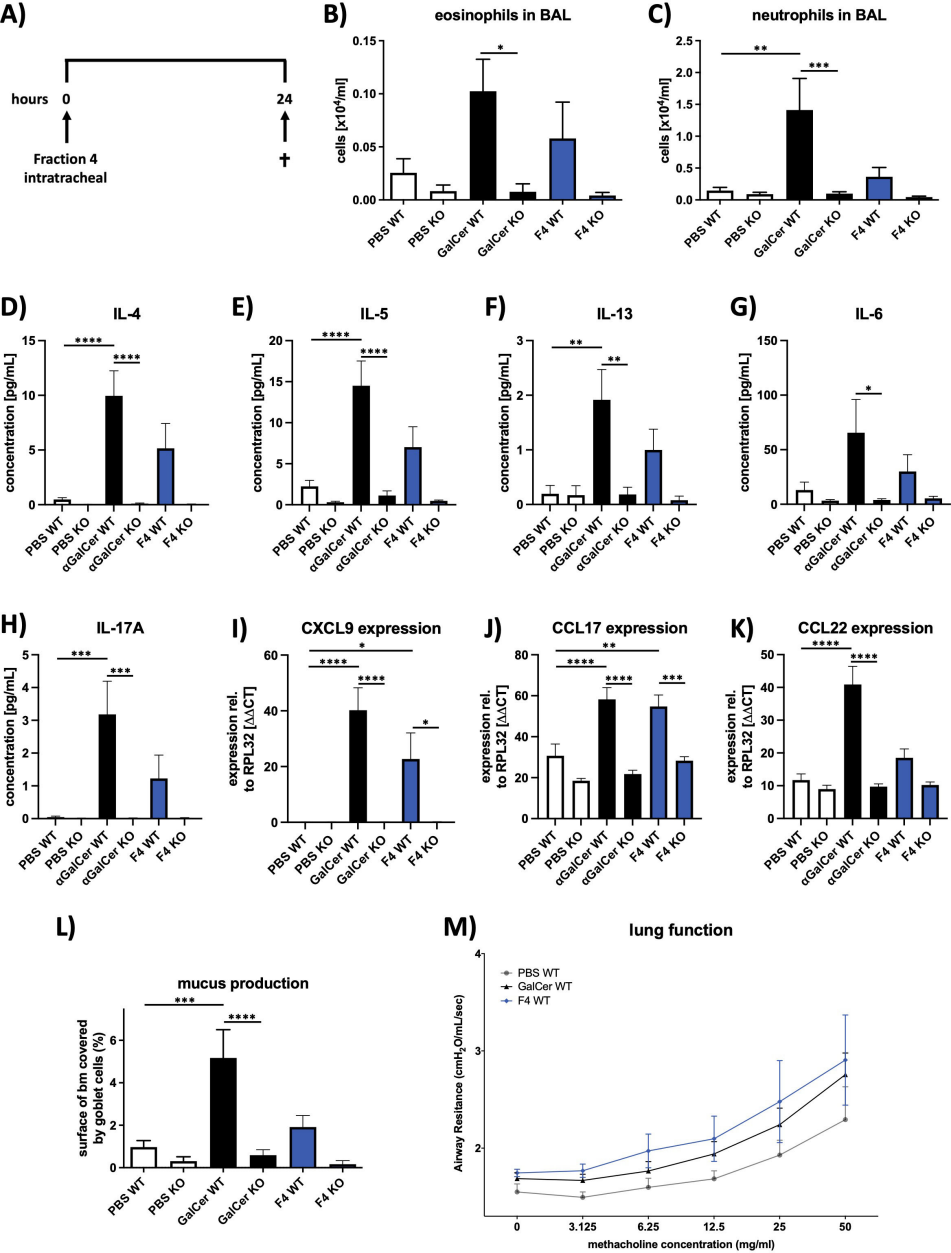
Fraction 4 induces airway inflammation and mucus production similar to αGalCer. **(A)** Treatment protocol for the *in vivo* mouse experiments. Mice were given fraction 4 intratracheally and killed 24 h later. Numbers of eosinophils **(B)** and neutrophils **(C)** in BALF of PBS-, αGalCer-, or F4-treated wild-type or Cd1d^−/−^ mice. **(D–H)** Concentrations of IL-4. IL-5, IL-13, IL-6, and IL-17A in BALF, assessed with MSD U-Plex assays. **(I–K)** After mRNA isolation, expression of the chemokines CXCL9, CCL17, and CCL22 was analyzed by qRT-PCR; Rpl32 was used as the housekeeping gene. **(L)** Area of the epithelial basal membrane covered by goblet cells. **(M)** Airway resistance in response to methacholine inhalation. Data are shown as mean ± SEM. *n* = 12. Statistical significance was assessed using repeated measure one-way analysis of variance and Tukey’s multiple comparison post hoc analyses, *p < .05; **p < .01, ***p < .001; ****p < .0001.

To evaluate the potential effects of fraction 4 on the formation of pathophysiologic features associated with allergic asthma, e.g., allergic airway inflammation, mucus hypersecretion, and airway hyperresponsiveness (AHR), mice received fraction 4, αGalCer, or PBS intratracheally and were analyzed 24 h later (**Figure 4A**). Indeed, animals revealed increased infiltration of eosinophils and neutrophils into the bronchoalveolar lumen after treatment with either fraction 4 or αGalCer (**Figures 4B, C**). Inflammation was associated with enhanced levels of typical T helper 2 (TH2) type cytokines interleukin (IL-)4, IL-5, and IL-13 (**Figures 4D–F**), as well as of proinflammatory IL-6 and IL-17A (**Figures 4G, H**) and chemokine CCL22 (**Figure 4K**). T helper cell, NK cell, and NKT cell attractant chemokines CXCl9 and CCL17 were significantly increased 24 h after a single dose of F4 (**Figures 4I, J**). Furthermore, animals treated with either fraction 4 or αGalCer displayed increased mucus production (**Figure 4L**) and increased airway reactivity to methacholine (**Figure 4M**), which, however, did not reach the level of significance. Remarkably, none of these features could be observed in CD1d-deficient mice.

### Fraction 4 contains a galactolipid

Since fraction 4 induced a stronger iNKT cell proliferation when compared to fraction 3, we proceeded with the preparative isolation of fraction 4, which would allow the subsequent isolation of pure glycolipid and its structure determination. A total of 10 mg of fraction 4 (0.2% of TG) yielded 16 fractions after RP18-HPLC. GC-MS analysis of these fractions revealed the presence of Glc, Gal, Inositol, Gro, 16:0, 18:0, 18:1, 18.2, 18:3, phytosphingosine and hydroxy fatty acids: 18:0(2-OH), 19:0(2-OH), 20:0(2-OH), and 22:1(2-OH) in fractions 8 (0.013% of TG), 9 (0.014% of TG), and 10 (0.008% of TG). These three fractions were combined and purified further by silica-HPLC, yielding phytoceramide (Glc, phytosphingosine, 18:1, 24:1(2-OH) and a galactolipid (Gal, Gro, 16:0, 18:2, 18:3). Since galactose-containing glycolipids are known to be potent NKT cell activators (20) we focused on the isolation of the galactolipid (0.005% of TG), determined its structure by ESI-MS (**Supplementary Figure S1**) and NMR (**Supplementary Table 1**; **Supplementary Figure S2**), which appeared to be 1,2-di-O-acyl-3-O-(alpha-D-Galp-1-6-beta-D-Glp)-sn-glycero (DGDG) as a mixture with five different combinations of fatty acids (1-*O*-sn-glycero/2-*O*-sn-glycero): 16:0/16:0 (DGDG-1), 16:0/18:3 (DGDG-2), 18:2/18:3 (DGDG-3), 18:3/18:2 (DGDG-4), and 18:3/18:3 (DGDG-5) (**Figure 1**).

All five variants of DGDGs were synthesized to avoid any potential environmental/microbial contamination of the isolated compounds, which could affect the biological activity, but more importantly, to have chemically defined DGDG molecules. The isolated fraction 4 and synthetic DGDG were subjected to *in vitro* and *in vivo* experiments.

### Differential immune activation by synthetic DGDG variants: selective induction of IL-13

To continue with the structure-function characterization of galactolipids present in fraction 4, we have synthesized all five variants of DGDG differentiated based on the acylation pattern, designated as DGDG-1, DGDG-2, DGDG-3, DGDG-4, and DGDG-5 (**Figure 2A**), to evaluate their biological activity. Splenocytes were incubated with each synthetic analog, αGalCer, or left untreated for 96 h, after which cell proliferation and cytokine production were assessed. Flow cytometry analysis revealed that DGDG-1, DGDG-2, and DGDG-3 induced a clear proliferative response, whereas DGDG-4 and DGDG-5 showed minimal to no activation (**Figures 2B, C**, left graph). Furthermore, while all DGDG variants induced the production of the Th1 cytokine IFN-γ, only DGDG-2 and DGDG-3 triggered the release of the Th2 cytokine IL-13 (**Figure 2C**, middle and right graphs), a cytokine implicated in allergic inflammatory responses such as asthma. These results suggest that specific DGDG acylation patterns may differentially regulate immune responses, particularly those associated with Th2-driven inflammation.

### Synthetic DGDG-1~3 induces inflammation and mucus production in the airways similar to fraction 4

**Figure 5:**
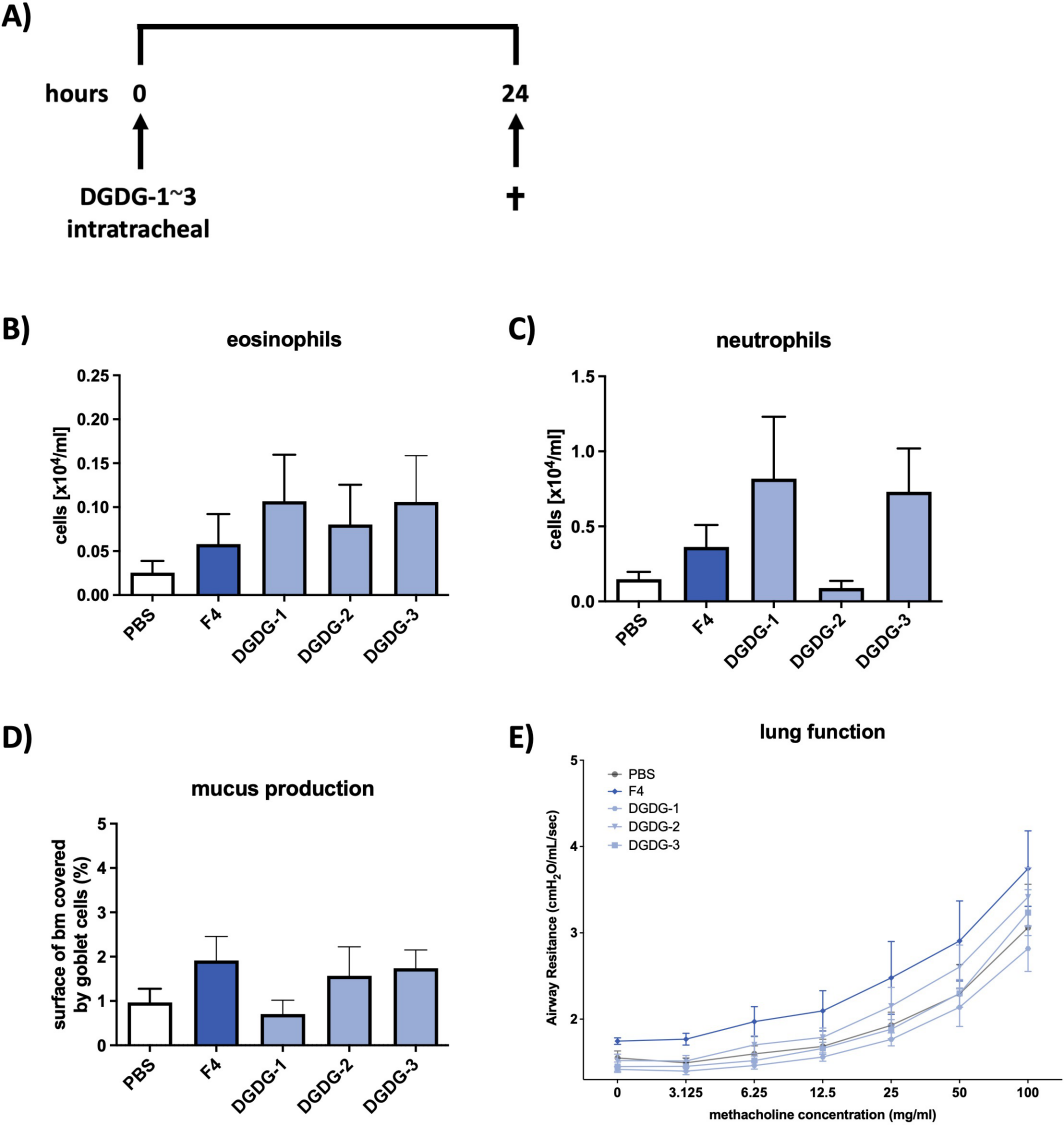
Synthetic DGDG-1~3 induces inflammation and mucus production in the airways similar to fraction 4. **(A)** Treatment protocol for the *in vivo* mouse experiments. Mice were treated intratracheally with DGDG-1~3 and killed 24 h later. Numbers of eosinophils **(B)** and neutrophils **(C)** in BALF of PBS, F4, or DGDG-1~3-treated wild-type mice. **(D)** Area of epithelial basal membrane covered by goblet cells. **(E)** Airway resistance in response to methacholine inhalation. Data are shown as mean ± SEM. *n* = 12.

Since DGDG-1~3 showed to be involved in the Th2 response *in vitro*, we wanted to investigate whether these specific variants are involved in the formation of the pathophysiologic hallmarks of asthma observed after treatment with fraction 4. Mice received either synthetic DGDG-1~3, fraction 4, or PBS and were analyzed 24 h later (**Figure 5A**). Indeed, similarly to fraction 4, treatment with either DGDG-1 or DGDG-3 led to increased infiltration of eosinophils and neutrophils into the bronchoalveolar lumen, whereas treatment with DGDG-2 led to increased infiltration of eosinophils only (**Figures 5B, C**). Additionally, fraction 4, the synthetic glycolipids DGDG-2 and DGDG-3, increased mucus production in the airways, whereas mucus production of DGDG-1-treated animals was at the level of PBS-treated animals (**Figure 5D**). In contrast to fraction 4, treatment with DGDG-1~3 did not lead to the development of AHR (**Figure 5E**).

## Discussion

Our work on the water-insoluble lipid components of allergenic grass pollen adds to the growing body of evidence that allergens alone are not sufficient to trigger allergic inflammation but that lipid components of the pollenkitt regulate and prepare the immune system for the allergen-antigen recognition (27, 28). The grass pollen particles containing allergens and lipids released after their rupture in the atmosphere are small enough to reach the lungs and encounter immune cells (29).

By applying ultrasonication to TG pollen during extraction, we disrupted the grains and thereby obtained a lipid composition representative of both the external and internal components of TG pollen grains (30). In this work, by using a targeted approach, we isolated galactolipids, which are the most prominent lipids in the plant membranes, with their fatty acids being commonly polyunsaturated of C16/C18 type (28). As such, di-galactosyl lipids are a rich source of α-linolenic acid (ALA, 18:3) (31). DGDGs are known to play a role in photosynthesis and chloroplast membrane structure, especially in thylakoid membranes (32). However, to date, nothing is known about the function of DGDG in allergic inflammation (28).

In our study, out of the five DGDG isolated from grass pollen and their corresponding synthetic analogs with a specific fatty acid composition, only (1-*O*-sn-glycero/2-*O*-sn-glycero): 16:0/16:0 (DGDG-1), 16:0/18:3 (DGDG-2), and 18:2/18:3 (DGDG-3) elicited immune responses *in vitro* and *in vivo*, with a cytokine production associated with Th2 polarization, such as IL-13, known to play a central role in allergic inflammation; whereas 18:3/18:2 (DGDG-4) and 18:3/18:3 (DGDG-5) did not induce a response. Hence, highlighting how small structural differences influence glycolipid recognition in a structure-function fashion (33). This response aligns with previously described pathways leading to the development of allergic asthma, where lipid antigens stimulate NKT cells and other immune subsets, resulting in eosinophilic infiltration and airway hyperreactivity (29). Previous studies have shown that glycolipids presented by CD1d molecules can act as antigens that modulate allergic responses, thus supporting the idea that DGDG may function similarly for immune cell activation towards an allergic phenotype (34, 35).

In this context, it is noteworthy that certain *Phleum pratense* allergens, such as Phl p 2 and Phl p 6, display hydrophobic properties that may facilitate interactions with lipid components like DGDGs during allergen delivery. While Phl p 5, a major allergen, lacks defined lipid-binding pockets, its structural hydrophobic regions may still support lipid association within the pollen matrix. Such co-delivery of allergens with lipid adjuvants could enhance antigen uptake and modulate immune activation via CD1d-restricted NKT cells, thereby influencing the sensitization process.

Plant glycolipids, predominantly glycoglycerolipids like DGDG, consist of a glycerol backbone linked to simpler sugar moieties such as galactose and often bear polyunsaturated fatty acids like linolenic acid (36, 37). Mammalian glycolipids, mainly glycosphingolipids such as cerebrosides and gangliosides, are based on a sphingosine backbone, have more complex sugar moieties (e.g., glucose, galactose, and sialic acid), and typically include long-chain saturated or unsaturated fatty acids (37, 38). The structural similarities between plant-derived glycolipids and those capable of modulating mammalian immune responses, namely the presence of a long hydrophobic lipid tail that anchors the glycolipid in the CD1d groove and a hydrophilic sugar head group that interacts with the TCR on NKT cells, suggest that the DGDG found in grass pollen may contribute to the immunogenicity of pollen allergens (27). This is supported by evidence that lipid metabolism and specific fatty acid compositions of galactolipids can influence the activation and differentiation of immune cells involved in allergic inflammation, such as Th2 cells, eosinophils, and mast cells (39–41).

Beyond plant-derived lipids, sphingolipids, such as ceramides and sulfatides, have also been shown to drive type-2 immune responses, particularly in asthma (42). These lipids activate iNKT cells, triggering eosinophilic lung infiltration and cytokines critical in asthma and allergic airway diseases (43). Structurally, the common feature shared by DGDG, αGalCer, and sphingolipids is the polar head group (in DGDG, a di-galactose structure) and the hydrophobic tail, both of which are essential for CD1d presentation and recognition by iNKT cells. This structural similarity likely allows DGDG to mimic the immune-stimulating effects of other glycolipids involved in type-2 inflammation (33).

Furthermore, other structural lipids, such as lysophosphatidylcholines (LPCs), are implicated in exacerbating allergic inflammation, hence adding supporting evidence to their potential role in type-2 immunity. LPCs activate innate immune cells like macrophages and dendritic cells, promoting the production of Th2 cytokines and exacerbating allergic responses (44). The galactose headgroup in DGDG may similarly interact with innate immune receptors, enhancing their ability to contribute to allergic inflammation, particularly in the lungs (45). This suggests that DGDG in grass pollen may serve as lipid antigens that contribute to pollen’s allergenicity—a hypothesis warranting further investigation.

Interestingly, only fraction 4, but none of the DGDG, led to a moderate increase in airway reactivity in our mouse model of allergic inflammation. The reasons why this increase does not reach the level of significance and thus the development of AHR remain to be answered. In patients with asthma as well as in mouse models of experimental allergic asthma, development of AHR is closely associated with the influx of eosinophils into the airway tissue and bronchoalveolar lumen, and eosinophils have been shown to contribute to the development of AHR by releasing not only an array of cationic proteins but also neurotrophins (46–48). Since the number of eosinophils in BALF of animals treated with fraction 4 (or also with DGDG-1~3) is significantly higher than in healthy controls, but is moderately increased as compared to established mouse models of experimental asthma, it seems reasonable that the amount of inflammation is not sufficient to induce AHR. Also, fraction 4 showed the strongest influence on mucus production as DGDG-1~3. This suggests the role of other components in fraction 4, such as phytoceramides, in contributing to these two characteristics of airway inflammation. To our knowledge, there are no reports on the effect of pollen-derived phytoceramides on allergic inflammation. However, due to the high similarity of phytoceramides to the prototypic ligand for NKT cells (49), αGalCer, we can speculate that phytoceramides have the potential to activate NKT cells, thus having a synergistic effect with DGDG, directing the immune status towards allergic inflammation. This hypothesis will be further tested.

## Conclusion

Taken together, our study is the first to describe, at the molecular level, how galactolipids interact with NKT cells and how their fatty-acid composition governs potency. Our findings also reveal a novel role for grass pollen-derived DGDGs in allergic responses, extending beyond their established structural functions in plants. These galactolipids may act as immunomodulators, contributing to the allergic inflammation observed in conditions like hay fever and asthma (50). Given that NKT cells can both enhance and regulate immune responses through cytokines like IL-13 and IFN-γ, their activation by pollen-derived lipids may influence not only the intensity but also the direction of the immune response.

Thus, we not only identified previously unrecognized immunogenic components of pollen but also introduced new potential therapeutic targets. Future interventions focusing on modulating lipid–allergen interactions could offer novel strategies to prevent allergic diseases, such as asthma and hay fever, by targeting the lipid components of environmental allergens rather than solely protein allergens.

The abundant presence of diverse lipid species in pollen cytoplasm highlights the need for further research to fully understand their impact on lung health. Evidence that small chemical variations significantly affect biological activity lays the foundation for the future development of personalized lipid-allergen formulations for allergen-specific immunotherapy (AIT).

## Data Availability

The datasets presented in this study can be found in online repositories. The names of the repository/repositories and accession number(s) can be found below: SUBSTANCE UPLOAD ID 128016 (Validated II) and ID 128018 (PubChem).
