# Supplementary material for: Phleum pratense pollen-derived di-galactosyldiacylglycerols promote pro-allergic responses in mice
Source: Front Immunol. 2025 Jun 17;16:1532773. doi: 10.3389/fimmu.2025.1532773 (PMC12208850; doi:10.3389/fimmu.2025.1532773)
Supplement: Supplementary file 1 [file DataSheet1.docx]

**ONLINE SUPPLEMENT**

**Supplementary Figures and Table**

**
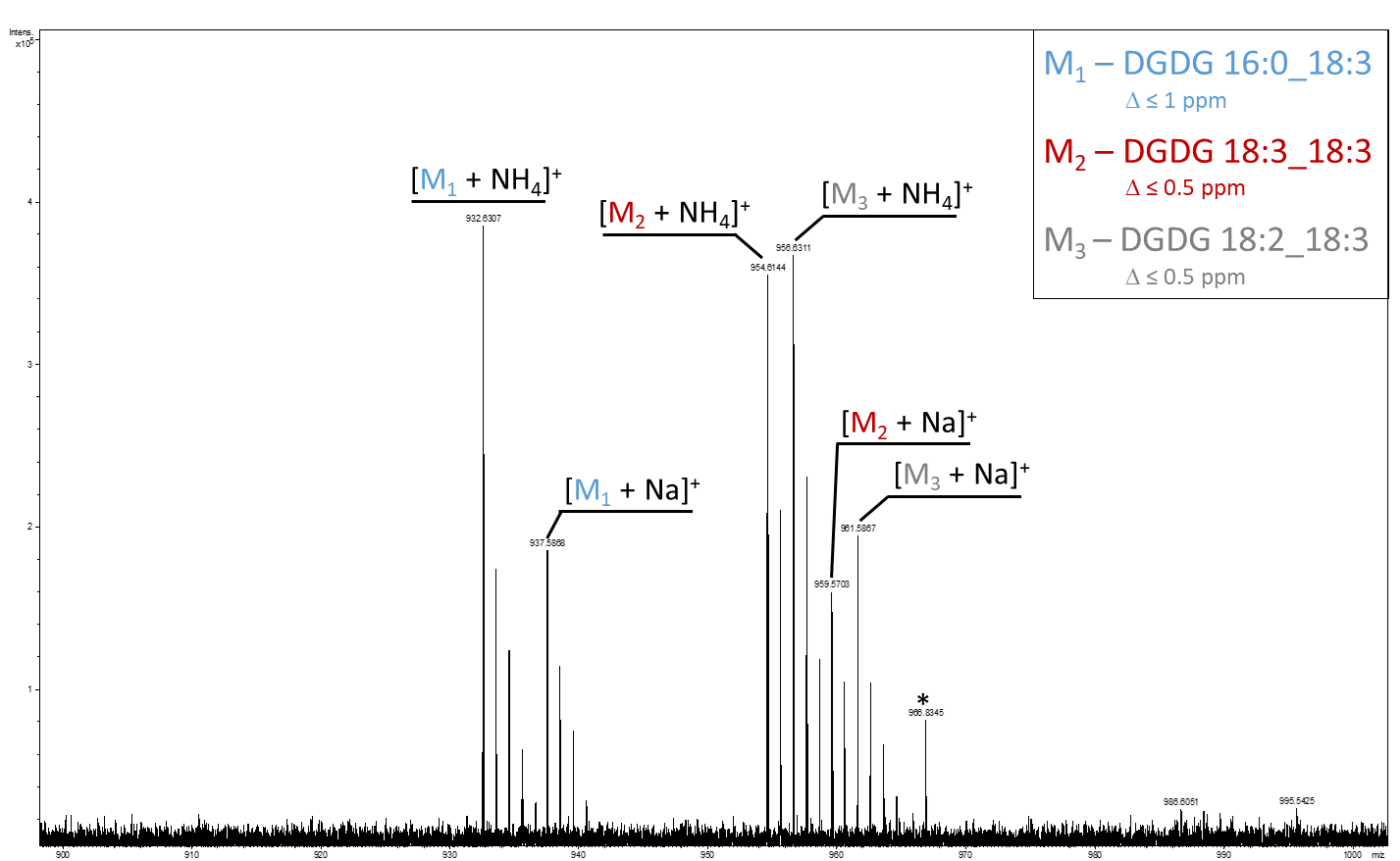
**

**Supl. Fig. 1:** Spectrum of positive nano-ESI MS of purified fraction 4 containing 3 DGDG species. DGDG lipids were detected as ammonium and sodium adducts with overlapping isotopic patterns (* spectral artifact).

**
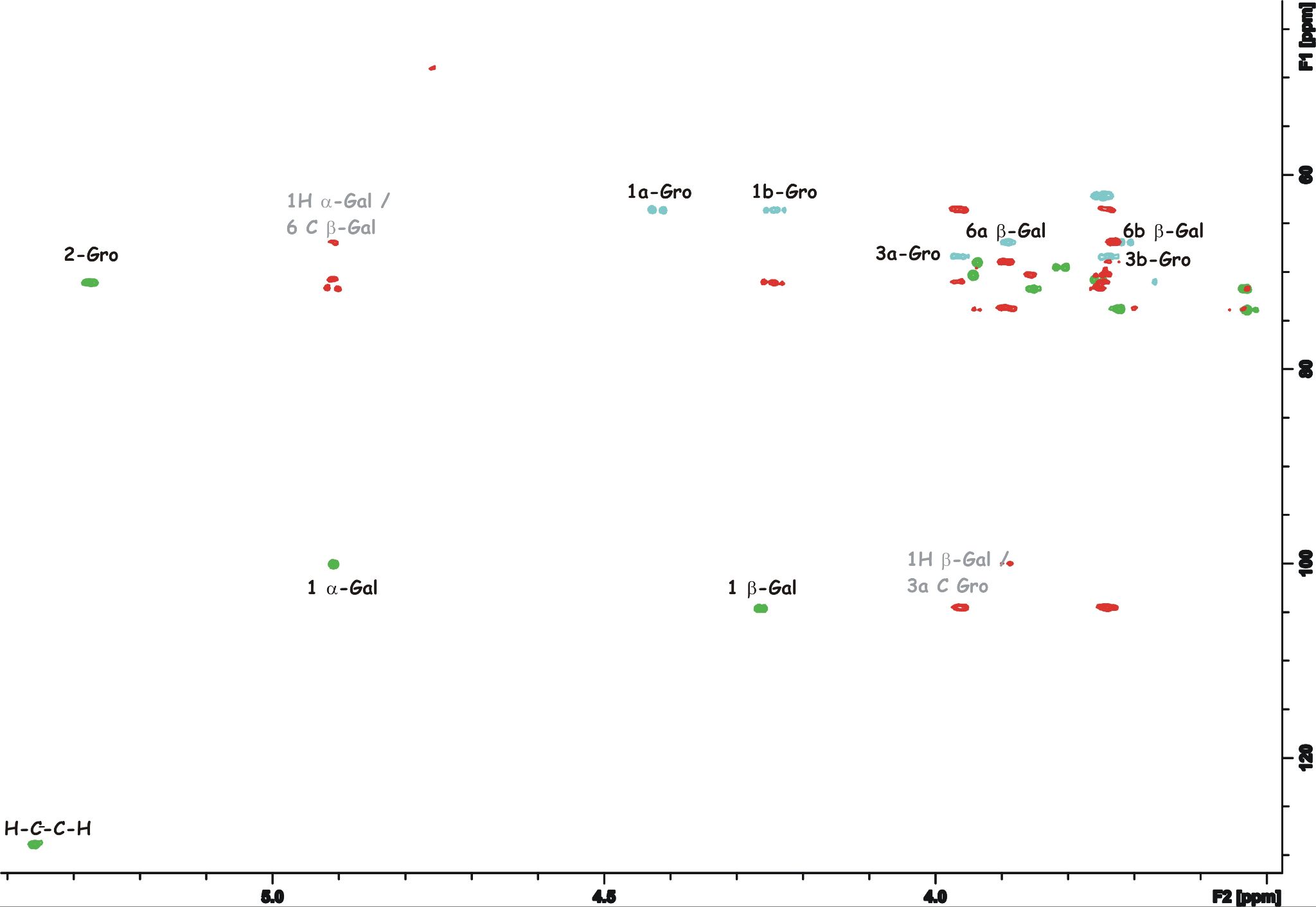
**

**Supl. Fig. 2:** Overlay of HSQC (green, blue) and HMBC (red) spectra of DGDG. Spectra were recorded at 27 °C in CDCl_3_/MeOH/D2O 40:53:7, 300 K and calibrated to TMS (d_H_ 0.0, d_C_ 0.0)

|  | | 1 (^3^*J*_1,2_) | 2 | 3 (^3^*J*_3,4_) | 4 | 5 | 6a | 6b |
| --- | --- | --- | --- | --- | --- | --- | --- | --- |
| a-d-Gal*p* | H | 4.91  (3.7 Hz) | 3.81 | 3.74 | 3.93 | 3.53 | 3.75 | 3.75 |
|  | C | 100.03 | 69.49 | 70.73 | 69.01 | 71.67 | 62.09 | |
| b-d-Gal*p* | H | 4.26 (6.9 Hz) | 3.52 | 3.93 | 3.85 | 3.73 | 3.90 | 3.71 |
|  | C | 104.65 | 73.87 | 70.30 | 71.71 | 73.76 | 66.90 | |
|  | | 1a | 1b | 2 | 3a | 3b |  |  |
| Gro | H | 4.42 | 4.24 | 5.27 | 3.96 | 3.74 |  |  |
|  | C | 63.62 | | 71.07 | 68.45 | |  |  |

**Table 1:** ^1^H and ^13^C NMR chemical shifts (∆d, p.p.m.) of DGDG.

Spectra were recorded at 27 °C in CDCl_3_/MeOH/D2O 40:53:7, 300 K and calibrated to TMS (d_H_ 0.0, d_C_ 0.0)
